# MicroPET Outperforms Beta-Microprobes in Determining Neuroreceptor Availability under Pharmacological Restriction for Cold Mass Occupancy

**DOI:** 10.3389/fnins.2017.00047

**Published:** 2017-02-10

**Authors:** Dorien Glorie, Stijn Servaes, Jeroen Verhaeghe, Tine Wyckhuys, Leonie Wyffels, Olivier Vanderveken, Sigrid Stroobants, Steven Staelens

**Affiliations:** ^1^Faculty of Medicine and Health Sciences, Molecular Imaging Center Antwerp, University of AntwerpAntwerp, Belgium; ^2^Nuclear Medicine Department, Antwerp University HospitalAntwerp, Belgium; ^3^Translational Neurosciences, Department of Otorhinolaryngology and Head and Neck Surgery, Faculty of Medicine and Health Sciences, University of Antwerp, Antwerp University HospitalAntwerp, Belgium

**Keywords:** μPET, beta-microprobe, rat brain, [^11^C]raclopride, [^11^C]ABP688

## Abstract

Both non-invasive micro-positron emission tomography (μPET) and *in situ* beta-microprobes have the ability to determine radiotracer kinetics and neuroreceptor availability *in vivo*. Beta-microprobes were proposed as a cost-effective alternative to μPET, but literature revealed conflicting results most likely due to methodological differences and inflicted tissue damage. The current study has three main objectives: (i) evaluate the theoretical advantages of beta-microprobes; (ii) perform μPET imaging to assess the impact of (beta-micro)probe implantation on relative tracer delivery (R1) and receptor occupancy (non-displaceable binding potential, BP_ND_) in the rat brain; and (iii) investigate whether beta-microprobe recordings produce robust results when a pharmacological restriction for cold mass dose (tracer dose condition) is imposed. We performed acquisitions (*n* = 61) in naive animals, dummy probe implanted animals (outer diameter: 0.75 and 1.00 mm) and beta-microprobe implanted animals (outer diameter: 0.75 mm) using two different radiotracers with high affinity for the striatum: [^11^C]raclopride (*n* = 29) and [^11^C]ABP688 (*n* = 32). In addition, acquisitions were completed with or without an imposed restriction for cold mass occupancy. We estimated BP_ND_ and R1 values using the simplified reference tissue method (SRTM). [^11^C]raclopride dummy μPET BP_ND_ (0.75 mm: −13.01 ± 0.94%; 1.00 mm: −13.89 ± 1.20%) and R1 values (0.75 mm: −29.67 ± 4.94%; 1.00 mm: −39.07 ± 3.17%) significantly decreased at the implant side vs. the contralateral intact side. A similar comparison for [^11^C]ABP688 dummy μPET, demonstrated significantly (*p* < 0.05) decreased BP_ND_ (−19.09 ± 2.45%) and R1 values (−38.12 ± 6.58%) in the striatum with a 1.00 mm implant, but not with a 0.75 mm implant. Particularly in tracer dose conditions, despite lower impact of partial volume effects, beta-microprobes proved unfit to produce representative results due to tissue destruction associated with probe insertion. We advise to use tracer dose μPET to obtain accurate results concerning receptor availability and tracer delivery, keeping in mind associated partial volume effects for which it is possible to correct.

## Introduction

Brain implants provide valuable information on the central nervous system. They are commonly used in neuroscience to deliver drugs by guided cannulas, perform neurochemical sampling through microdialysis probes, stimulate brain tissue with deep brain stimulation electrodes, or measure radiotracer kinetics using beta-microprobes. However, these research tools are unavoidably invasive as implant diameters frequently exceed the dimensions of brain tissue components (neurons, capillaries, and fiber bundles), thereby destroying the integrity of the tissue. Acute implantation causes changes in the local cerebral blood flow, metabolism, and neurotransmitter concentration (Grabb et al., 1998; Mauger et al., 2005), possibly confounding collected measurements.

Currently, two preclinical methods are available to determine radiotracer kinetics and neuroreceptor occupancy *in vivo*: *in situ* beta-microprobes and non-invasive micro-positron emission tomography (μPET). These techniques provide the opportunity to measure neuroreceptor changes, associated with neurodegenerative or psychiatric disease. Following a stereotaxic implantation in the rat brain, radiosensitive beta-microprobes can measure the local concentration of a radiolabeled molecule within a few millimeters around its tip. This technique proved useful for different purposes; such as cerebral blood flow measurements (Weber et al., 2003), metabolic studies (Moulin-Sallanon et al., 2005), radiotracer evaluation (Wyss et al., 2007), arterial input function measurements (Warnock et al., 2011), and drug challenge experiments (Ginovart et al., 2004). μPET offers a non-invasive alternative, by visualizing radiotracer distribution in the entire brain and enabling longitudinal studies through intra-animal comparisons. Although, incapable of providing information on spatial tracer distribution (Märk et al., 2013), beta-microprobes are suggested as a cost-effective alternative to μPET. They possess a higher temporal resolution (Zimmer et al., 2002) to estimate the peak of a time-activity curve, higher sensitivity (Pain et al., 2008), lower partial volume effect (Ginovart et al., 2004), and easier coupling with other *in vivo* techniques (Desbrée et al., 2004). In addition, beta-microprobes offer the possibility to perform measurements in awake animals without restraint combined with behavioral observations (Balasse et al., 2014). To ensure representative results, μPET and beta-microprobe experiments should comply with low mass tracer dose (TD) conditions. For TD conditions, the injected mass dose remains sufficiently low to preserve normal physiology (Hume et al., 1998; Madsen et al., 2011) with a maximal receptor binding of 5–10% by the biomarker.

Previously, literature conveyed conflicting results (Zimmer et al., 2002; Ginovart et al., 2004) on beta-microprobe robustness and signal-to-noise ratio, probably due to differences in scintillating tip size (sensitivity), outer diameter (OD) tightly linked with tissue trauma, injected mass dose, and methodology. Hence, our aim was to rigorously compare high temporal resolution yet invasive *in situ* beta-microprobes vs. whole brain μPET to evaluate neuroreceptor binding and relative tracer delivery *in vivo*. If we assume that beta-microprobes are a viable alternative to μPET, this might pave the way for a wider use of beta-microprobes. Beta-microprobes are more easily accessible compared to μPET, which is associated with relatively high costs and specialized facilities.

First, we assessed the impact of implantation and probe OD on tracer delivery and receptor occupancy by inserting 0.75 and 1.00 mm OD dummy probes in the striatum and cerebellum. Beside, we aimed to clarify whether beta-microprobes provide a viable alternative to μPET, when TD is a strict boundary condition. To answer this question, we performed beta-microprobe acquisitions with the administration of either a TD or high dose (HD) for a subsequent comparison to μPET acquisitions. To conduct these experiments, we chose two different radiotracers with a high affinity for the striatum: [^11^C]raclopride ([^11^C]RAC) for the dopamine D2 receptor (Alexoff et al., 2003) and [3-(6-methyl-pyridin-2-ylethynyl)-cyclohex-2-enone-0-^11^C-methyloxime] ([^11^C]ABP688) for the metabotropic glutamate receptor 5 (Ametamey et al., 2007). The cerebellum, characterized by low specific binding of both radiotracers, suits as a reference region (Wu and Carson, 2002; Elmenhorst et al., 2010, 2012). After a volume-of-interest (VOI) analysis of the striatum and cerebellum, we estimated the non-displaceable binding potential (BP_ND_) in the striatum and the ratio of tracer delivery to the striatum (tissue of interest) relative to the delivery in the cerebellum (reference tissue; R1) using the simplified reference tissue method (SRTM; Lammertsma and Hume, 1996) for both μPET and beta-microprobe acquisitions. These physiologically relevant output parameters provide information on regional receptor availability (BP_ND_) and perfusion/transport of the radiotracer to the tissue of interest (R1).

## Materials and methods

### Study design

**Figure 1:**
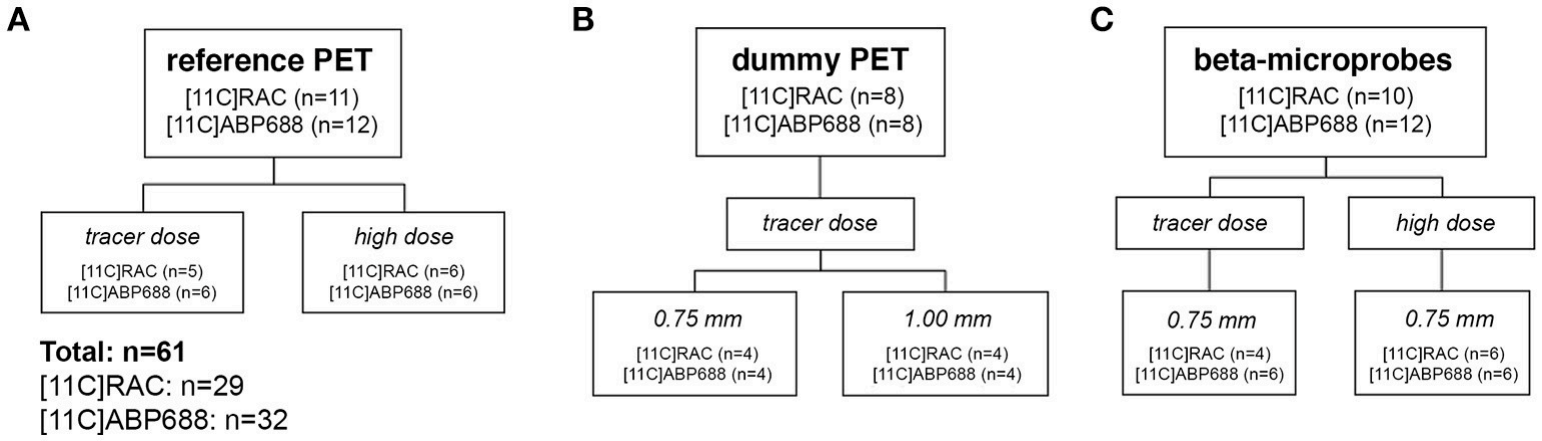
**Study design showing the number of animals in all experimental groups for both [^11^C]RAC and [^11^C]ABP688. (A)** μPET scans including low mass tracer dose (TD) and high dose (HD) acquisitions in animals without brain implants as a reference. **(B)** Assess the impact of the (dummy) implant OD (0.75 vs. 1.00 mm) on radiotracer kinetics and binding using μPET. **(C)** Clarify whether beta-microprobes provide a viable alternative for μPET, with TD as a boundary condition. This experiment included TD and HD acquisitions and was carried out using the 0.75 mm OD beta-microprobes. High dose (HD), ±65 MBq; μPET, micro positron emission tomography; low mass tracer dose (TD), [^11^C]RAC <0.5 nmol/kg /[^11^C]ABP688 <3.0 nmol/kg.

Male Sprague–Dawley rats (*n* = 61, Harlan Laboratories), weighing 364 ± 38 g, were treated in accordance with the European Ethics Committee (decree 86/609/CEE). The study protocol was approved by the Animal Experimental Ethical Committee of the University of Antwerp, Antwerp, Belgium. The animals were kept in individually ventilated cages under controlled conditions (12 h normal light–dark cycles, 20–23°C, and 50–55% relative humidity) with water and rodent food pellets *ad libitum*. The study design (Figure 1) was identical for both radiotracers ([^11^C]RAC: *n* = 29 − [^11^C]ABP688: *n* = 32). For each radiotracer the design is subdivided in three separate experimental groups: a reference μPET group without implants; a dummy implant μPET group; and a beta-microprobe 0.75 mm OD implant group. Further subdivisions were made based on different experimental conditions such as injected dose (TD vs. HD) and implant diameter. All animals received either a high dose (target dose of 65 MBq; cf. Table 1) or a low mass tracer dose being lower than 0.5 nmol/kg (Schiffer et al., 2005) for [^11^C]RAC and lower than 3.0 nmol/kg (Wyckhuys et al., 2013) for [^11^C]ABP688. Every radiotracer administration was combined with either a reference μPET-CT scan without implantations (Figure 1A), a dummy probe implant combined with a μPET-CT scan (Figure 1B), or a beta-microprobe implant and acquisition (Figure 1C). The dummy μPET experimental group was further subdivided in separate implant groups depending on the OD and thus the inflicted tissue trauma (**Figure 3**). These dummy groups were mandatory since simultaneous μPET and beta-microprobe acquisitions are practically unfeasible as the actual scintillation probes are non-disposable and cannot be permanently fixed with dental cement (cf. Section Experimental Procedure). Three out of 64 subjects (*n* = 61) were left out for further analysis due to surgical complications (bleeding) or when the maximal allowed cold mass was exceeded for TD conditions.

**Table 1:** **Average (±s.d.) specific activity (SA) at the time of injection, injected dose (ID), and injected mass (IM) for all experimental groups**.

	**Animals** ***n***	**SA (MBq/nmol)** **Average ± s.d**.	**ID (MBq)** **Average ± s.d**.	**IM (nmol/kg)** **Average ± s.d**.
**[^11^C]RAC**				
Reference μPET HD	6	93.15 ± 51.86	73.35 ± 1.98	2.86 ± 1.78
Reference μPET TD	5	18.45 ± 4.51	3.31 ± 1.51	0.44 ± 0.11
Dummy μPET TD 0.75 mm	4	57.63 ± 11.65	9.72 ± 2.17	0.48 ± 0.01
Dummy μPET TD 1.00 mm	4	51.26 ± 10.93	9.32 ± 1.23	0.50 ± 0.01
Beta-microprobe HD 0.75 mm	6	65.45 ± 46.07	67.08 ± 8.13	4.77 ± 3.06
Beta-microprobe TD 0.75 mm	4	38.49 ± 10.98	7.24 ± 0.50	0.52 ± 0.03
**[^11^C]ABP688**				
Reference μPET HD	6	48.77 ± 15.97	69.81 ± 3.45	4.48 ± 2.01
Reference μPET TD	6	17.34 ± 3.66	20.81 ± 5.63	2.90 ± 0.32
Dummy μPET TD 0.75 mm	4	18.53 ± 2.26	16.82 ± 1.12	2.72 ± 0.09
Dummy μPET TD 1.00 mm	4	17.97 ± 2.70	16.40 ± 0.50	2.73 ± 0.07
Beta-microprobe HD 0.75 mm	6	47.18 ± 24.55	65.68 ± 3.33	5.60 ± 3.93
Beta-microprobe TD 0.75 mm	6	16.81 ± 1.10	15.32 ± 2.34	2.52 ± 0.33

*HD, high dose; ±65 MBq; ID, injected dose; IM, injected mass; PET, positron emission tomography; SA, specific activity; s.d., standard deviation; low mass tracer dose (TD), [*^11^*C]RAC <0.5 nmol/kg /[*^11^*C]ABP688 <3.0 nmol*

### Experimental procedure

For the reference μPET-CT scans (Figure 1A), the animals were anesthetized (2% isoflurane mixed with medical oxygen). After the insertion of a catheter in the tail vein for tracer administration, the animals were positioned on the heated bed (37.5°C) of the μPET-CT scanner.

For beta- and dummy probe implantations (Figures 1B,C), the animal was anesthetized (2% isoflurane mixed with medical oxygen) ~90 min before radiotracer administration and placed on a heated blanket (37.5°C). Before surgery, a catheter was placed in the tail vein for tracer administration. Subsequently, the head of the animal was fixated in a stereotaxic frame (Kopf Instruments, California, USA). After making an anteroposterior incision toward the neck region, the skull was exposed by retraction of the skin and muscle layers. Two holes were drilled in the skull for probe positioning, one at the level of the right striatum and another at the level of the cerebellum. The anteroposterior (AP) and mediolateral (ML) stereotaxic coordinates were determined relative to the bregma. The surface of the dura functioned as a reference point for the dorsoventral (DV) coordinate. Implant coordinates were obtained from a rat brain atlas (Paxinos and Watson, 2013): AP +0.2, ML 3.0, DV −6.0 for the striatum and AP −11.8, ML 0.0, and DV −3.5 for the cerebellum. Finally, the dummy probes (0.75 mm or 1.00 mm OD) or beta-microprobes (0.75 mm OD) were positioned. Beta-microprobe animals remained on the heated blanket and were coupled to the light-tight Beta Scintillator System Twin (Swisstrace, Zurich, Switzerland; Weber et al., 2003; Wyss et al., 2007), awaiting immediate tracer administration. Dummy probe animals were transferred to the μPET-CT scanner (Siemens Preclinical Solution, Knoxville, TN, USA) after fixation of the dummy probes on the skull with dental cement (prorep propal, Belgica Dental, Belgium).

Concerning the synthesis of both radiotracers, [^11^C]RAC was synthesized by O-methylation of (S)-O-desmethylraclopride precursor (ABX, Raderberg, Germany) with ^11^C-CH_3_O_3_SCH_3_. ^11^C-CO_2_ was produced in an Eclips HP cyclotron (Siemens, Knoxville, TN, USA) by irradiation of a N_2_ target for 50 min with an 11 MeV proton beam and a beam intensity of 60 μA. The ^11^C-CO_2_ was converted to ^11^C-CH_3_O_3_SCH_3_ for reaction with (S)-O-desmethylraclopride via ^11^C-CH_3_I. The radiochemical purity was determined by reverse phase high-performance liquid chromatography (HPLC) (Waters Symmetry C18, 3.5 μm, 4.6 × 50 mm) and was ≥99.9%. The purified [^11^C]RAC was dissolved in a mixture of NaH_2_PO_4_ 0.005M pH 4.5, 0.9% NaCl (B. Braun, Germany) and ethanol 96% v/v (BP, Eur.Ph., Merck-Chemicals, Darmstadt, Germany). The solution was sterile filtered for *in vivo* use. [^11^C]ABP688-E is prepared by reaction of 0.5 mg desmethyl-ABP688 (E/Z) with ^11^C-CH_3_SO_3_CF_3_ in 400 μL acetone in the presence of 10 μL 1N NaOH for 4 min at room temperature. For purification, the crude reaction mixture was injected into an analytical HPLC column (WatersXBridge C18, 5 μm, 4.6 × 150 mm) with an eluent of 0.05 M sodium acetate (pH 5.5) and ethanol (55/45, v/v) at a flow rate of 1 mL/min to isolate the [^11^C]ABP688-E. The purified [^11^C]ABP688-E is filtrated through a sterile Millipore Millex-GV filter (0.22 μm) and diluted with 0.9% NaCl through the same filter to reduce the ethanol concentration to <10%. The average specific activity, injected dose, and injected mass dose for all experimental groups are shown in Table 1. In order to keep the injected mass dose below the imposed limit of 0.5 nmol/kg for [^11^C]RAC and 3.0 nmol/kg for [^11^C]ABP688, injected doses in the TD groups accurately compensate for the varying specific activity of each radiotracer production.

### Data acquisition

The reference μPET and the dummy implant groups received a dynamic μPET-CT scan (Figures 1A,B) performed on two Siemens Inveon μPET-CT scanners (Siemens Preclinical Solution, Knoxville, TN, USA) with the following specifications: 60-min dynamic μPET acquisitions with 2 × 10 s, 3 × 20 s, 3 × 30 s, 3 × 60 s, 3 × 150 s, 9 × 300 s frames, followed by a 7-min μCT. Scans were started immediately after positioning the animal on the scanner and administration of a bolus of radiotracer via the tail vein. All animals were randomly distributed on both scanners.

For beta-microprobe recordings (Figure 1C), the light-tight Beta Scintillator System Twin was switched on at least 30 min before recording to ensure a sufficient warm-up and stabilization of the photomultiplier tubes (Ginovart et al., 2004). The system was equipped with 0.75 mm OD scintillator probes. After implantation, recordings at a sampling rate of 1 Hz were started 1 min before tracer administration for count stabilization followed by a 60-min acquisition using PMOD v3.0 (PMOD technologies, Zurich, Switzerland). Finally, the animals were removed from the stereotaxic frame and the scintillator probes were cleansed with water. All animals were injected with an overdose of pentobarbital (>30 mg/kg) at the end of each acquisition (beta-microprobe and dummy μPET). Upon immediate removal, their brain was submerged in cooled 2-methylbutane and stored at −20°C for a subsequent histological verification of the probe position in coronal cryosections (20 μm). Cryosections confirmed correct dummy probe localization for all subjects.

Both μPET scanners were calibrated using a uniform cylinder with known activity (±37 MBq; room temperature) to obtain a calibration factor for conversion of images to kBq/cc. Calibration (sampling rate: 1 Hz; duration: 60 s) of beta-microprobes was achieved by immersion in an aqueous solution (10 mL) with a known activity concentration of [^11^C]ABP688 (±20 MBq; room temperature). Probes were repetitively calibrated (similar experimental conditions) in order to determine an averaged calibration factor for each probe.

### Data analysis

For quantitative image analysis, μPET images were reconstructed using two-dimensional ordered subset expectation maximization with four iterations and 16 subsets after Fourier rebinning. The images were reconstructed on a 128 × 128 × 159 grid with a voxel size of 0.776 × 0.776 × 0.796 mm. Normalization, dead time correction, random subtraction, CT-based attenuation correction, and single-scatter simulation scatter corrections were applied. For both tracers, reconstructed images were processed in PMOD v3.3. A static image corresponding to the time-averaged frames of each dynamic acquisition was spatially transformed through brain normalization to a rat brain template for [^11^C]RAC and [^11^C]ABP688 (in-house developed; Verhaeghe et al., 2014). Both PET templates already corresponded to a standardized MR template space (Schiffer rat MR; available in PMOD v3.3) with corresponding VOI definition. The obtained matrix from the brain normalization step was applied to transform all dynamic scans to the [^11^C]RAC and [^11^C]ABP688 template space, respectively. The time-activity curves of the striatum and cerebellar VOI were extracted from the resulting images via the superimposition of a VOI template. The extracted time-activity curves served as input for the SRTM (Lammertsma and Hume, 1996). This kinetic modeling method allows quantification of receptor kinetics in a region of interest via the expression of tracer uptake in this tissue in terms of its uptake in a reference region (devoid of the receptor-of-interest) with a similar level of non-specific binding. Based on this method (which is implemented in PMOD software), the BP_ND_ and relative delivery R1 of the tracer to the striatal VOI were calculated, with the cerebellum as a reference region. In addition, pixel wise kinetic modeling, using SRTM2 (Wu and Carson, 2002), was applied to generate parametric BP_ND_ and R1 maps.

For each beta-microprobe experiment, the recorded time-activity curves of the striatum and cerebellum were decay corrected for ^11^C with respect to the timing of radiotracer injection (PMOD v3.5). The individual probe sensitivity, represented by a mean calibration factor for each probe, was applied to convert measured counts per second (cps) into radioactive concentration (kBq/cc) (PMOD v3.5). The resulting time-activity curves were resampled to μPET frame duration (2 × 10 s, 3 × 20 s, 3 × 30 s, 3 × 60 s, 3 × 150 s, 9 × 300 s) to reduce the increasing statistical counting noise, arising from the rapid decay of ^11^C. The corrected time-activity curve data points for both probes (striatum and cerebellum) were fitted using the SRTM to obtain the BP_ND_ and R1 parameters for individual measurements.

Data are expressed as the averaged BP_ND_ and R1 ± standard error of the mean (s.e.m.) for each experimental group. The statistical significance of these SRTM output values (striatum) for both μPET and beta-microprobe experiments was evaluated using a non-parametric Mann–Whitney test in GraphPad Prism 6 (GraphPad Software, San Diego, USA). Differences were considered statistically significant at *p* < 0.05.

## Results

### Radiotracer dosing impacts the degree of receptor occupancy reflected by the non-displaceable binding potential (BP_ND_) parameter

**Figure 2:**
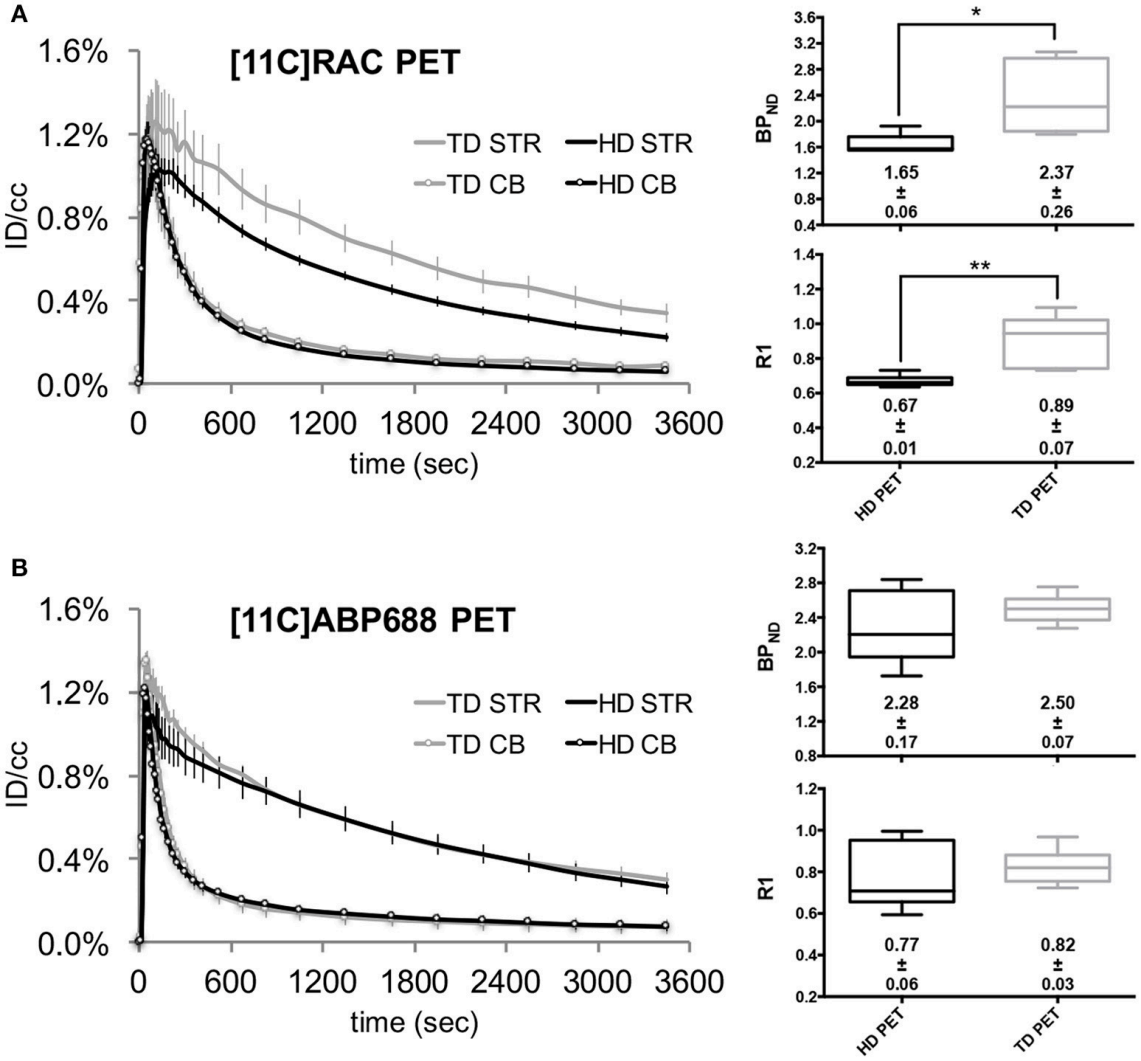
**Averaged injected dose corrected time-activity curves (±s.e.m.) with the corresponding BP_ND_ and R1 values for [^11^C]RAC and [^11^C]ABP688 reference HD and TD μPET experiments**. The left panels show the averaged injected dose corrected time-activity curves for HD (±65 MBq) vs. TD ([^11^C]RAC: <0.5 nmol/kg; [^11^C]ABP688: <3.0 nmol/kg) μPET experiments, for [^11^C]RAC **(A)** and [^11^C]ABP688 **(B)**, respectively. The average BP_ND_ and R1 values (±s.e.m.) are displayed with the corresponding box-plot (right panels). The asterisks indicate significant (*p* < 0.05) differences in BP_ND_ or R1 values after a non-parametric Mann–Whitney test. CB, cerebellum; HD, high dose ±65 MBq; ID/cc, injected dose/cubic centimeter; PET, positron emission tomography; STR, striatum; TD, low mass tracer dose [^11^C]RAC <0.5 nmol/kg /[^11^C]ABP688 <3.0 nmol/kg.

When comparing two dose conditions (TD and HD) using reference (without implant) μPET scans, the HD, and TD cerebellar time-activity curves both show the characteristic temporal profile of a reference region with a fast decline toward a low level (±0.1%ID/cc) for both radiotracers (Figure 2). Evidently, the corresponding striatal time-activity curves differ in temporal profile: the HD striatal time-activity curves visibly decline to a lower level for both tracers compared to the TD time-activity curves. This “dose effect” is more pronounced for the [^11^C]RAC striatal time-activity curves (Figure 2A) reflected by the corresponding BP_ND_ values (Figures 2A,B box-plots): a HD (±65 MBq) administration of [^11^C]ABP688 caused a 8.94% (±0.33%) decrease in the striatal BP_ND_ compared with TD (<3.0 nmol/kg) μPET experiments (Figure 2B). A similar HD BP_ND_ decrease is more pronounced for [^11^C]RAC (−30.48 ± 2.04%), which reached significance (*p* = 0.0173; Figure 2A).

### Histology confirms a disproportional impact of probe outer diameter on tissue damage

**Figure 3:**
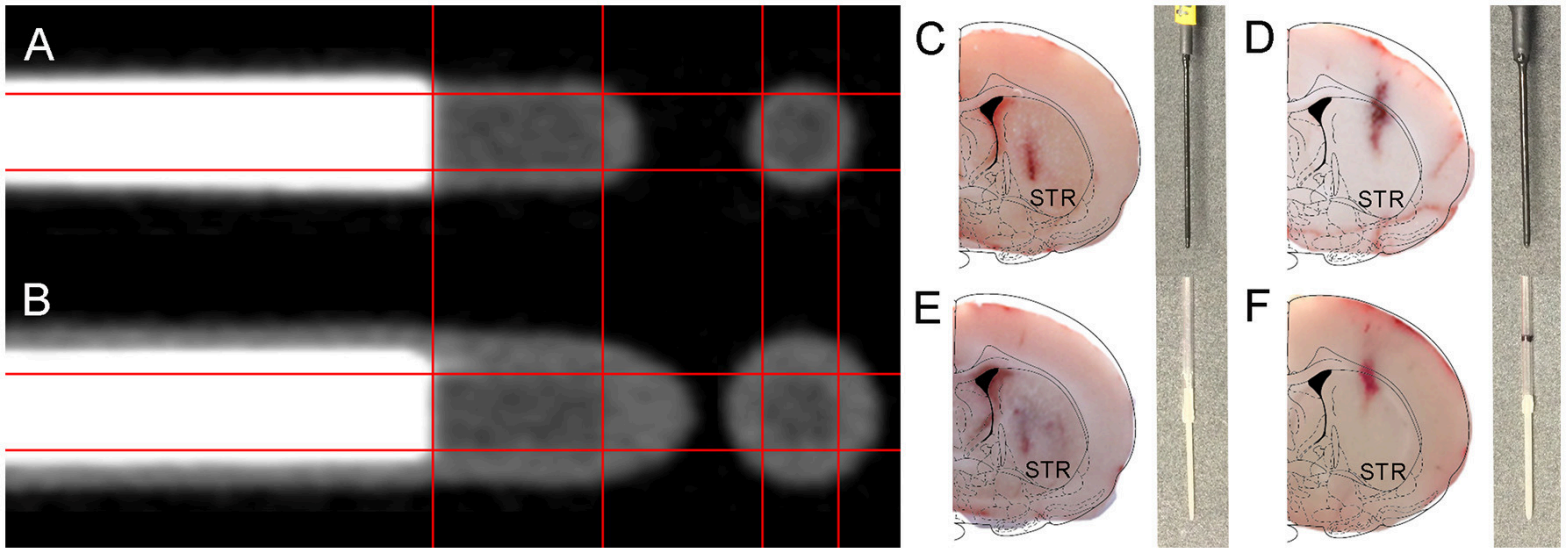
**Illustration of the internal composition of the available beta-microprobe sets and inflicted implant damage to the rat brain. (A,B)** High resolution μCT-images from **(A)** 0.75 mm OD and **(B)** 1.00 mm OD beta-microprobes. **(C–F)** Results from the histological verification of probe localization in coronal cryosections of the striatum (indicated by STR; brain outline adapted from Paxinos and Watson) (Paxinos and Watson, 2013). The gray insets show the corresponding implanted probe for each cryosection: **(C)** 0.75 mm beta-microprobe; **(D)** 1.00 mm beta-microprobe; **(E)** 0.75 mm dummy probe; **(F)** 1.00 mm dummy probe.

High-resolution μCT images (Figures 3A,B) confirm similar scintillating tip dimensions (1.00 mm length–0.50 mm diameter) in both beta-microprobe sets, which results in a similar sensitivity with however a difference in outer diameter (0.75 and 1.00 mm) by additional coating. Cryosections show tissue damage with both beta-microprobe sets (Figures 3C,D) disproportionally increasing with outer diameter. Additionally, our in-house dummy probes for μPET imaging were designed to have a similar shape and size (Figures 3E,F) and proved suitable to mimic the inflicted damage from beta-microprobe implantations, for both the 0.75 mm (Figure 3C) and 1.00 mm (Figure 3D) OD beta-microprobe set.

### Probe implantation affects both radiotracer perfusion and receptor occupancy

**Figure 4:**
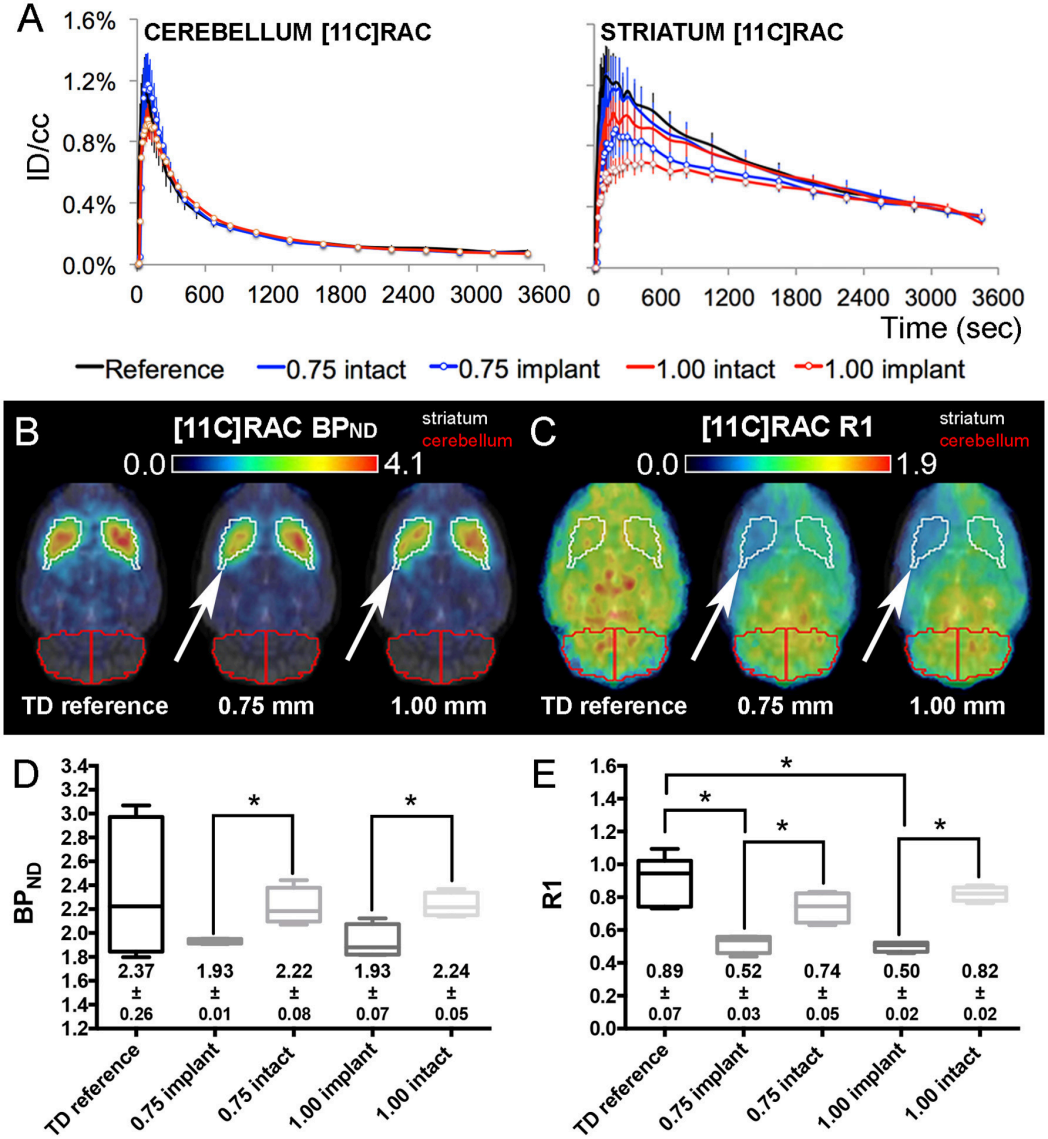
**[^11^C]RAC average injected dose corrected time-activity curves (±s.e.m.) combined with the corresponding BP_ND_ and R1 maps/values from TD reference and dummy μPET experiments. (A)** [^11^C]RAC reference and dummy (0.75 and 1.00 mm) μPET time-activity curves (±s.e.m.) for the cerebellum (reference region) and the striatum (region of interest). Dummy groups received a probe implantation in both the cerebellum and striatum in contrast to the reference group. **(B,C)** Average transversal BP_ND_ **(B)** and R1 **(C)** maps for the reference, 0.75 mm OD dummy, and 1.00 mm OD dummy μPET groups overlaid on a magnetic resonance (MR) template. The white arrows point out the side of dummy implantation. **(D,E)** Box-plot representation of the BP_ND_ **(D)** and R1 **(E)** values obtained from TD μPET experiments (with and without dummy implants). The average BP_ND_ and R1 values (±s.e.m.) are displayed at the bottom of the corresponding graph. The asterisks indicate significant (*p* < 0.05) differences in BP_ND_ or R1 values using a non-parametric Mann–Whitney test. Remark: the time-activity curves from [^11^C]RAC reference TD μPET acquisitions (Figure 2A) were repeated here for a sufficient comparison of data. BP_ND_, non-displaceable binding potential; ID/cc, injected dose/cubic centimeter; PET, positron emission tomography; R1, relative delivery; TD, low mass tracer dose <0.5 nmol/kg.

**Figure 5:**
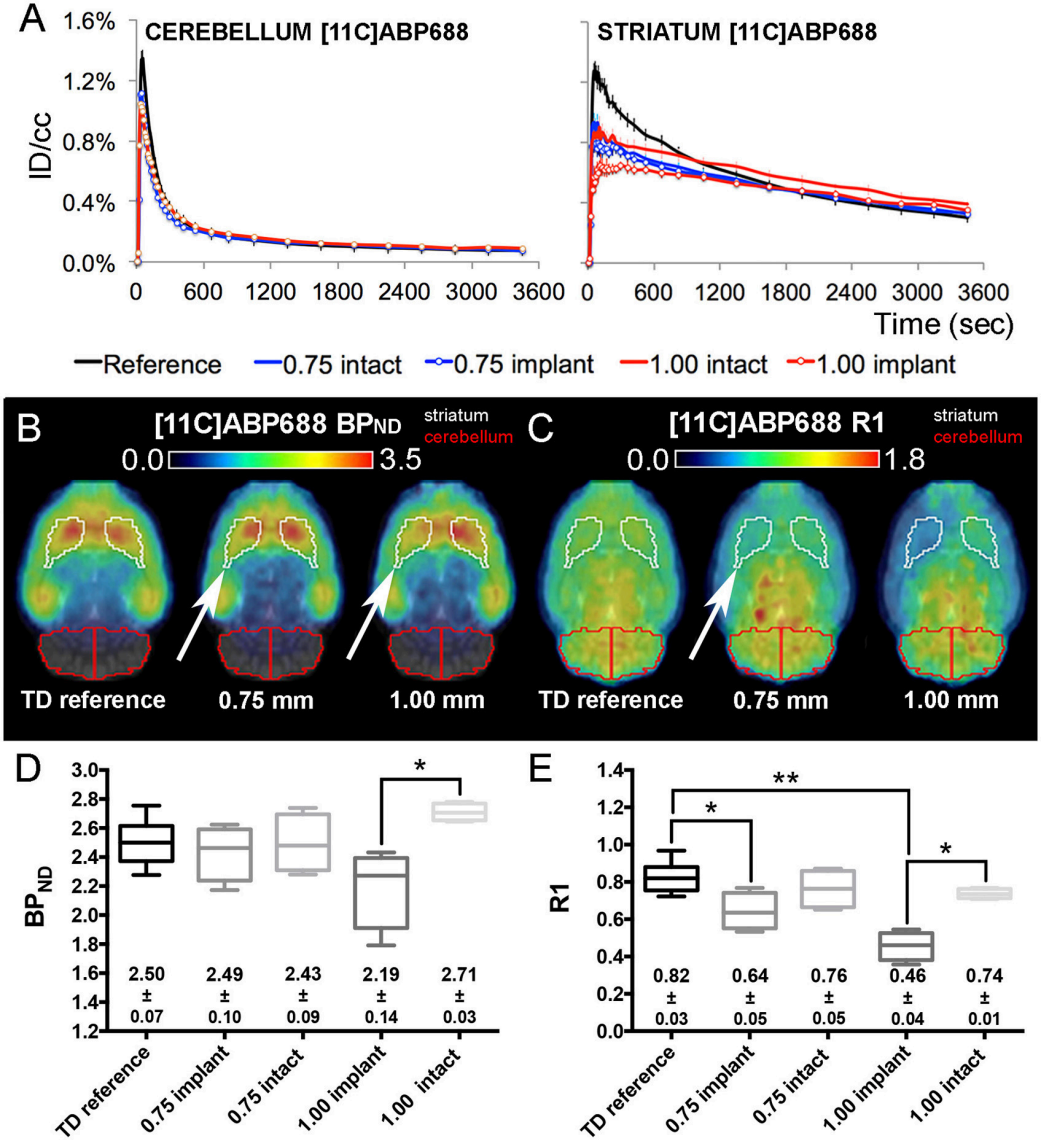
**[^11^C]ABP688 average injected dose corrected time-activity curves (±s.e.m.) combined with the corresponding BP_ND_ and R1 maps/values from TD reference and dummy μPET experiments. (A)** [^11^C]ABP688 reference and dummy (0.75 and 1.00 mm) μPET time-activity curves (±s.e.m.) for the cerebellum (reference region) and the striatum (region of interest). Dummy groups received a probe implantation in both the cerebellum and striatum in contrast to the reference group. **(B,C)** Average transversal BP_ND_ **(B)** and R1 **(C)** maps for the reference, 0.75 mm OD dummy, and 1.00 mm OD dummy μPET groups overlaid on a magnetic resonance (MR) template. The white arrows point out the side of dummy implantation. **(D,E)** Box-plot representation of the BP_ND_ **(D)** and R1 **(E)** values obtained from TD μPET experiments (with and without dummy implants). The average BP_ND_ and R1 values (±s.e.m.) are displayed at the bottom of the corresponding graph. The asterisks indicate significant (^*^p < 0.05/^**^p < 0.01) differences in BP_ND_ or R1 values using a non-parametric Mann–Whitney test. Remark: the time-activity curves from [^11^C]ABP688 reference TD μPET acquisitions (Figure 2B) were repeated here for a sufficient comparison of data. BP_ND_, non-displaceable binding potential; ID/cc, injected dose/cubic centimeter; PET, positron emission tomography; R1, relative delivery; TD, low mass tracer dose <3.0 nmol/kg.

Figure 4A ([^11^C]RAC) and Figure 5A ([^11^C]ABP688) illustrate the corresponding time-activity curves for the cerebellum and the striatum of the reference μPET as well as the TD μPET experiments for both the 0.75 and 1.00 mm OD dummies compared to the intact hemisphere. The striatal time-activity curves of dummy implanted rats differed in temporal profile compared to reference μPET striatal time-activity curves and compared to the intact contralateral side, especially at the level of the peak and significantly more pronounced for 1.00 mm dummy probes.

In both [^11^C]RAC dummy groups, when comparing the implant (white arrows) and intact striatal VOIs, the BP_ND_ maps (Figure 4B) and R1 maps (Figure 4C) show significant (*p* < 0.05) decreases in BP_ND_ (0.75 mm: −13.01 ± 0.94%; 1.00 mm: −13.89 ± 1.20%) and R1 values (0.75 mm: −29.67 ± 4.94%; 1.00 mm: −39.07 ± 3.17%) at the implant side (Figures 4D,E). Also, the averaged R1 values demonstrated significant (*p* < 0.05) reductions at the implant side for both dummy groups relative to the averaged reference μPET R1 value (Figure 4E).

For [^11^C]ABP688, a comparison between both sides (Figures 5B,C) revealed significantly (*p* < 0.05) decreased BP_ND_ (−19.09 ± 2.45%) and R1 values (−38.12 ± 6.58%) at the implant side of the 1.00 mm dummy group (Figures 5D,E). Also, the R1 values of the striatal VOIs with implant (Figure 5E) significantly differed (0.75 mm: *p* < 0.05 and 1.00 mm: *p* < 0.01) from the averaged reference TD μPET R1 value (0.82 ± 0.03). For [^11^C]ABP688, the BP_ND_ seems to be less influenced by the 0.75 mm implants.

### Tracer dose beta-microprobe acquisitions: not a viable alternative to tracer dose μPET

**Figure 6:**
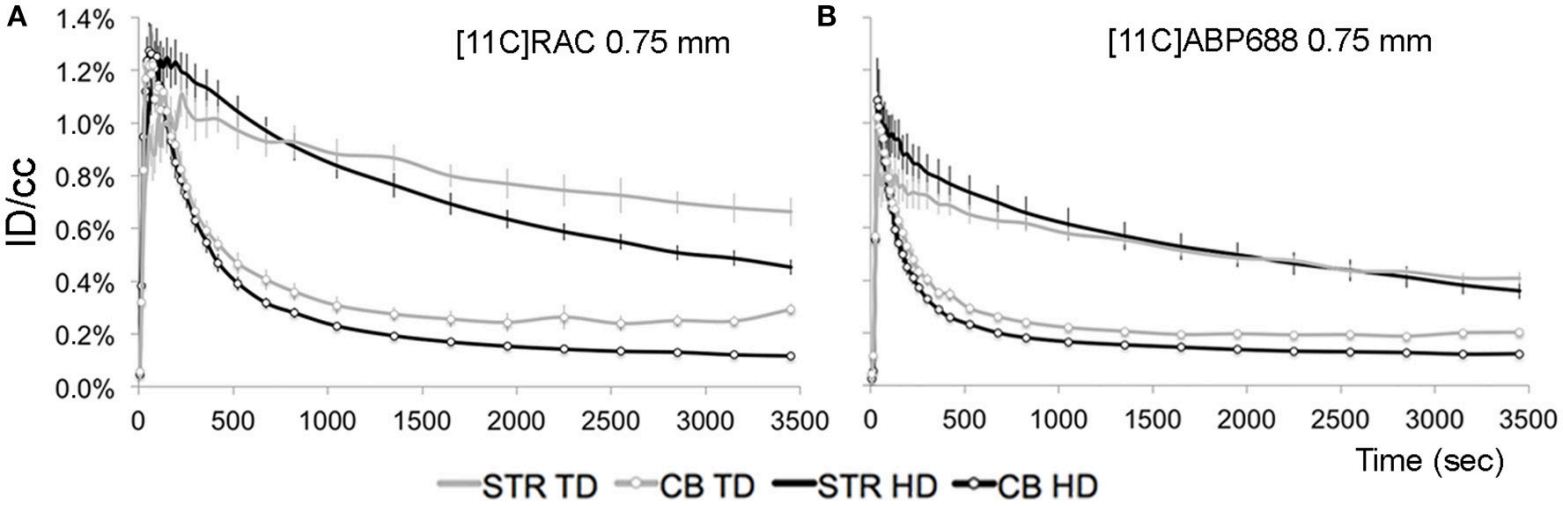
**Average injected dose corrected time-activity curves (±s.e.m.) measured by a beta-microprobe in a high-binding region (striatum) and reference region (cerebellum) after [^11^C]RAC or [^11^C]ABP688 administration. (A)** [^11^C]RAC striatal (without markers) and cerebellar (with markers) time-activity curves from 0.75 mm OD beta-microprobe measurements using varying dose conditions (TD vs. HD). **(B)** [^11^C]ABP688 striatal (without markers) and cerebellar (with markers) time-activity curves from 0.75 mm OD beta-microprobe measurements using varying dose conditions (TD vs. HD). CB, cerebellum; HD, high dose ±65 MBq; ID/cc, injected dose/cubic centimeter; STR, striatum; TD, low mass tracer dose [^11^C]RAC<0.5 nmol/kg /[^11^C]ABP688<3.0 nmol/kg.

Based on the results of the previous section, the 0.75 mm OD beta-microprobe set was selected for beta-microprobe recordings. The averaged and injected dose corrected time-activity curves for HD and TD beta-microprobe acquisitions are illustrated in Figure 6 for both [^11^C]RAC and [^11^C]ABP688. The corresponding averaged BP_ND_ and R1 values are shown in Table 2.

**Table 2:** **[^11^C]RAC and [^11^C]ABP688 average (±s.e.m.) striatal BP_ND_ and R1 values from HD and TD dose beta-microprobe measurements using a 0.75 mm OD beta-microprobe set**.

	**Animals** ***n***	**BP_ND_ striatum** **Average ± s.e.m**.	**R1 striatum** **Average ± s.e.m**.
**[^11^C]RAC BETA-MICROPROBE**			
HD 0.75 mm	6	2.12 ± 0.23	0.80 ± 0.08
TD 0.75 mm	4	1.65 ± 0.12	0.65 ± 0.08
**[^11^C]ABP688 BETA-MICROPROBE**			
HD 0.75 mm	6	2.12 ± 0.25	0.93 ± 0.13
TD 0.75 mm	6	1.31 ± 0.10	0.72 ± 0.06

*BP_ND_, non-displaceable binding potential; HD, high dose ±65 MBq; R1, relative delivery; s.e.m., standard error of the mean; TD, low mass tracer dose [*^11^*C]RAC<0.5 nmol/kg/[*^11^*C]ABP688 <3.0 nmol/kg*.

For TD recordings, the tail of the cerebellar reference curve distends (±3000 s) upon decay correction. A subsequent application of the SRTM to TD data resulted in inaccurate outcome parameters, producing a lower BP_ND_ value (Table 2) compared with HD recordings and conflicting with the reference μPET experiment (Figure 2).

HD recordings, where TD conditions are exceeded, yield lower average BP_ND_ values (Table 2) compared to reference TD μPET (Figure 2).

## Discussion

In the present study, we (i) evaluated theoretical advantages associated with beta-microprobe recordings, (ii) assessed the impact of implantation and probe OD on relative tracer delivery and receptor occupancy using reference and dummy μPET scans, and (iii) addressed whether recordings with beta-microprobes produce representative results when TD is imposed as boundary condition. Beta-microprobe recordings proved unfit to produce representative results, primarily due to the destructive effect of inserting a probe in brain tissue, especially in the context of low counts associated with the administration of a low mass TD.

The reference striatal BP_ND_ values from our TD μPET experiments in intact animals agree very well with literature for both [^11^C]RAC (Hoekzema et al., 2010; Topping et al., 2010) and [^11^C]ABP688 (Elmenhorst et al., 2012). The collection of accurate ligand-receptor binding data—avoiding BP_ND_ underestimation—requires the amount of cold ligand to remain below an imposed limit, insuring 5–10% receptor occupancy (TD conditions; Hume et al., 1998; Kung and Kung, 2005). The application of such a limit alludes to eliminate a possible disruption of normal physiology. In general, a higher amount of cold stable ligand lowers the opportunity for the hot ligand to bind its target. For [^11^C]RAC, an additional drug effect from the radiotracer itself on presynaptic dopamine receptors could induce extra endogenous dopamine release, contributing to such underestimation (Morris et al., 2014). For [^11^C]ABP688, the effect of endogenous glutamate levels on [^11^C]ABP688 binding in the rat brain remains controversial (Wyckhuys et al., 2013; Zimmer et al., 2015). Our data (Figure 2) show a correlation between the extent of BP_ND_ underestimation and exceeding the TD limit (based on the affinity of the tracer for its target) as the application of the same HD (±65 MBq) violates TD conditions with a factor 6.50 for [^11^C]RAC (<0.5 nmol/kg) and with a factor 1.54 for [^11^C]ABP688 (<3.0 nmol/kg; Table 1). Consequently, HD administration produces a more pronounced effect on [^11^C]RAC time-activity curves reflected by a larger BP_ND_ underestimation, as clearly illustrated by Figure 2.

A major theoretical advantage of beta-microprobes includes their high temporal resolution to capture the peak of the time-activity curve. However, during time-activity curve pre-processing and in accordance with literature (Pain et al., 2002; Zimmer et al., 2002; Ginovart et al., 2004; Moulin-Sallanon et al., 2005; Märk et al., 2013; Balasse et al., 2014), we were forced to resample the curves to larger time frames due to high signal noise associated with beta-microprobe recordings. We resampled the data to μPET frames: 2 × 10 s, 3 × 20 s, 3 × 30 s, 3 × 60 s, 3 × 150 s, 9 × 300 s. In this way, the beneficial theoretical temporal resolution of 1 s was lost.

Although, both systems were calibrated accurately, the [^11^C]RAC HD striatal time-activity curve of the beta-microprobe recording is of similar shape but proportionally higher compared to the HD striatal time-activity curve of μPET acquisitions over its entire course. This results in a higher BP_ND_ value. More specifically, the HD beta-microprobe striatal time-activity curve peaks at ±1.3% ID/cc and decreases to ±0.5% ID/cc (Figure 6A) at the end of the acquisition, while the HD μPET striatal curve peaks at ±1.0% ID/cc and ends at ±0.2% ID/cc (Figure 2A). This comparison between the [^11^C]RAC HD striatal time-activity curves of both methods points out an underestimation of [^11^C]RAC binding by μPET, caused by spill-out of the striatal VOI (partial volume effect). This phenomenon could not be deduced from [^11^C]ABP688 HD striatal time-activity curves for both methods. We suggest the tracer-specific distribution pattern as a possible cause. [^11^C]ABP688 binding is characterized by spill-in from surrounding brain regions in the striatal volume of interest, causing an artificial increase in the HD μPET striatal curve (Figure 2B). Thereby, balancing the existing striatal spill-out of the tracer. This contrasts with [^11^C]RAC, which only binds striatal dopamine D2 receptors and consequently is not subjected to spill-in signal from surrounding tissues (devoid of D2 receptors). Indeed, as visible on the obtained BP_ND_ maps and as anticipated, [^11^C]RAC binding (Figure 4B) was restricted to the striatum, whereas [^11^C]ABP688 (Figure 5B) also binds regions surrounding the striatum.

The impact of probe implantation on measurements is detrimental for beta-microprobe data. μPET TD acquisitions with dummy probes in both the striatum and cerebellum confirmed a disruptive effect on local striatal receptor binding (BP_ND_) and -to a larger extent- on relative tracer delivery to the implant region (R1). This differs from previous findings showing that insertion of a microdialysis probe (0.34 mm OD) in the striatum does not significantly influences local [^11^C]RAC receptor binding (Schiffer et al., 2005, 2006). We suggest two possible explanations for this finding: (i) This may be attributable to the relatively large OD of the inserted probe (0.75 mm) in beta-microprobe studies, which is in line with the established relation between increasing OD and relative tracer delivery. (ii) In contrast to the aforementioned studies where μPET images were acquired 2 days post implantation, we assessed implant effects immediately upon insertion (mimicking a typical beta-microprobe acquisition) thereby excluding possible adaptation mechanisms. In addition, one previous study revealed a 60% decrease in blood flow to the implant region after insertion of a 0.30 mm OD implant (Benveniste et al., 1987). Due to the dynamic relation between cerebral blood flow and drug delivery to the brain, we suggest a similar influence on tracer delivery to the damaged implant area, reflected by a visible decrease in the R1 parameter at the implant side. Subsequently, such decrease in R1 is inextricably linked to a decrease in BP_ND_.

Another phenomenon which possibly contributes to erroneous SRTM estimates for implanted animals is the possible presence of blood-brain barrier damage. The SRTM assumes an equal K1/k2 and K1′/k2′ for both the target and reference region(s). This implies an intact blood-brain barrier for both regions. Based on possible differences in the extent of blood-brain barrier damage between both regions, this assumption could become invalid thereby affecting the validity of SRTM. Additionally, we found erroneous SRTM estimates (BP_ND_ and R1) for beta-microprobe TD recordings due to a distorted cerebellar time-activity curve. During pre-processing, the applied ^11^C decay correction on this reference curve caused the tail to distend. This effect is more pronounced for [^11^C]RAC compared to [^11^C]ABP688 recordings, likely caused by the reliability of the reference region as the cerebellum is completely devoid of dopamine D2 receptors, but yet contains a small amount of metabotropic glutamate receptor 5. However, also for TD [^11^C]ABP688 beta-microprobe data our group (Wyckhuys et al., 2013) previously showed that beta-microprobes underestimated the striatal BP_ND_. The aforementioned problem with the cerebellar reference curve did not occur in previously published beta-microprobe studies with [^11^C]RAC using the SRTM, because all these studies substantially exceeded the upper limit for cold mass of 0.5 nmol/kg or so-called TD condition (Zimmer et al., 2002; Ginovart et al., 2004; Balasse et al., 2014). And indeed, the time-activity curves of our HD [^11^C]RAC beta-microprobe recordings, are consistent with such high activity curves (Balasse et al., 2014), peaking and declining to similar levels of %ID/cc. Therefore, we conclude that -to the best of our knowledge- the vast majority of beta-microprobe literature involves the application of a HD, thereby perturbing physiology causing underestimation of the BP_ND_.

In contrast to a theoretically expected higher BP_ND_ value for TD beta-microprobe measurements compared to TD μPET acquisitions, we found the opposite. We suggest that, although beta-microprobes suffer less from partial volume effects, the implant effect drastically distorts acquired results, especially in low signal-to-noise measurements with cold mass restriction. This was confirmed by the beta-microprobe data where the implant effect showed less pronounced upon injection of a HD, most likely due to the presence of sufficient counts, despite the damage. However, to obtain representative results (especially in rodents) TD conditions should always be applied, which in our hands resulted in erroneous SRTM estimates when using beta-microprobes. Also, the use of a beta-microprobe with a smaller OD and consequently lower associated damage would not provide a solution due to the trade-off between tip dimensions and sensitivity.

In conclusion, beta-microprobes are not preferred due to the decisive and disruptive effect of probe insertion on delivery and binding of a radiotracer to a target tissue. Keeping in mind the occurrence of partial volume effects and the absence of a golden standard, we suggest to use TD μPET to produce reliable results. This approach offers the additional opportunity to perform longitudinal study designs, thereby repetitively acquiring whole brain information without the need to sacrifice the animal.

## Author contributions

LW supervised the radiotracer productions; StSt designed the study and assisted in image analysis and manuscript drafting; DG executed the experimental design, analyzed the PET, and beta-microprobe data and drafted the manuscript; OV audited the surgical procedures and contributed to the manuscript draft; and SiSt proofread the manuscript. All authors approved the final manuscript and agree to be accountable for all aspects of the work.

## Funding

This work was funded by Antwerp University, Belgium through a Ph.D. grant for DG and StSe, an assistant professor position for JV and LW, an associate professor position for OV and StSt and a full professor position for SiSt. LW, OV, and SiSt are also supported by Antwerp University Hospital, Belgium through a departmental position. Experimental costs were supported by a University of Antwerp research contract (46/FA02006/WP130001).

### Conflict of interest statement

The authors declare that the research was conducted in the absence of any commercial or financial relationships that could be construed as a potential conflict of interest. The reviewer YS and handling Editor declared their shared affiliation, and the handling Editor states that the process nevertheless met the standards of a fair and objective review.
